# The effects of *Artemisia absinthium *L*.* on scopolamine-induced learning and memory impairment and brain tissue oxidative damage in adult rats

**DOI:** 10.22038/AJP.2022.62851.2991

**Published:** 2023

**Authors:** Marzieh Rahimi, Narges Marefati, Farimah Beheshti, Somaieh Ahmadabady, Hassan Rakhshandeh, Mahmoud Hosseini

**Affiliations:** 1 *Student Research Committee, Faculty of Medicine, Mashhad University of Medical Sciences, Mashhad, Iran*; 2 *Department of Physiology and Medical Physics, Faculty of Medicine, Baqiyatallah University of Medical Sciences, Tehran, Iran*; 3 *Neuroscience Research Center, Torbat Heydariyeh University of Medical Sciences, Torbat Heydariyeh, Iran*; 4 *Department of Physiology, School of Paramedical Sciences, Torbat Heydariyeh University of Medical Sciences, Torbat Heydariyeh, Iran*; 5 *Applied Biomedical Research Center, Mashhad University of Medical Sciences, Mashhad, Iran*; 6 *Pharmacological Research Center of Medicinal Plants, Mashhad University of Medical Sciences, Mashhad, Iran*; 7 *Psychiatry and Behavioral Sciences Research Center, Mashhad Universityof Medical Sciences, Mashhad, Iran*

**Keywords:** Artemisia absinthium, Memory, Oxidative stress, Scopolamine

## Abstract

**Objective:**

The present study examined the effects of *Artemisia absinthium* L. on scopolamine-induced memory dysfunction and brain tissue oxidative damage in rats.

**Materials and Methods:**

Fifty rats were used in five groups: Control: received dimethyl sulfoxide (DMSO)/saline, Scopolamine: scopolamine (2 mg/kg) was administered along with DMSO/saline, and Scopolamine-Ext 50, Scopolamine-Ext 100, and Scopolamine-Ext 200 groups: *A. absinthium* hydroalcoholic extract 50, 100 and 200 mg/kg were administered before scopolamine. The Morris water maze (MWM) and passive avoidance (PA) tasks were used for assessment of behavioral parameters. Malondialdehyde (MDA), nitric oxide (NO) metabolites, total thiol, catalase (CAT), and superoxide dismutase (SOD) were measured in the cortex and hippocampus.

**Results:**

*A. absinthium* decreased the delay time and distance traveled to reach the platform in the MWM test (p<0.05-p<0.001). Besides, the extract increased the delay time to pass in the dark and the light time while decreasing the number of entrances and the dark time in the PA task (p<0.05-p<0.001). In biochemical assessments, *A. absinthium* attenuated NO metabolites (p<0.001) and MDA (p<0.05- p<0.001) while enhanced total thiol (p<0.001), CAT and SOD (both p<0.05-p<0.001).

**Conclusion:**

This study revealed that *A. absinthium* improved memory and learning impairment and brain tissue oxidative damage in scopolamine-treated rats.

## Introduction

Dementia is a term that refers to a deterioration in mental functions that affects memory, cognition, or other mental abilities. As a result, occupational performance and social interactions may be impaired (Jafarian et al., 2019 ▶). The hippocampus, cortical and limbic brain regions are implicated in high-level brain functions, including learning, memory, perception, consciousness and self-awareness (Parihar et al., 2004 ▶). In the pathogenesis of neurodegenerative disorders, a state of oxidative stress coupled with neuroinflammation has been involved (Abareshi et al., 2004 ▶; Bargi et al., 2017 ▶). In comparison to other tissues, the brain and nervous system are more susceptible to oxidative damage because of the high proportion of polyunsaturated lipids present in neural parenchyma, high oxygen demand, and low levels of antioxidative enzymes (Fukui et al., 2001 ▶). Various brain regions are affected by oxidative damage leading to memory dysfunction, morphological defects and long-term complications (Parihar et al., 2004 ▶). The hippocampus is one of the main targets in neurodegenerative diseases, which is particularly sensitive to oxidative stress caused by free radicals, which manifests in accelerated lipid peroxidation and altered free radical defense systems (Bastianetto et al., 1999 ▶). Oxidative stress impairs the synaptic plasticity of the hippocampal formations and leads to cognitive deficits accompanied by impairment in daily functioning and memory loss (Postu et al., 2019 ▶; Rivas-Arancibia et al., 2010 ▶). 

In learning and memory processes, acetylcholine plays a crucial role. Impaired function of the cholinergic system has been linked to cognitive deficits and memory loss in neurodegenerative disorders (Beheshti et al., 2021 ▶). Scopolamine, as a non-selective muscarinic antagonist, binds to the muscarinic receptor with a high affinity and activates acetylcholinesterase in the cortex and hippocampal areas. As a result of the cholinergic hypofunction, it decreases cerebral blood flow and increases oxidative stress and inflammation in the brain, particularly in the hippocampal regions. Therefore, scopolamine contributes to dementia by impairing learning and memory in animals and humans. In this manner, an intraperitoneal (i.p.) administration of scopolamine provides an appropriate animal model for the investigation of memory deficit and the efficacy of potential therapeutics (Zhang et al., 2022 ▶).

*Artemisia absinthium *L*.* (family: Asteraceae), or wormwood, is a shrub-like perennial herb widely grown in the Middle East, Asia, Europe, and North Africa (Batiha et al., 2020 ▶). *A. absinthium* has numerous pharmacological activities, including neuroprotective (Bora and Sharma, 2010 ▶), hepatoprotective (Amat et al., 2010 ▶), antipyretic (Bora and Sharma, 2010 ▶), antidepressant (Mahmoudi et al., 2009 ▶), antibacterial (Dvorkin-Camiel and Whelan, 2008 ▶), and anti-inflammatory (Hadi et al., 2014 ▶) activities. Additionally, it has a wide range of antioxidant (Msaada et al., 2015 ▶) properties.* A. absinthium *exhibits potent free-radical scavenging capacity *in vitro* and *in vivo*, which may be correlated with phenols and flavonoids content of the extract (Bora and Sharma, 2011 ▶). The flavonoids, including rutin and quercetin, have been demonstrated to interact with signaling pathways and inhibit oxidative stress (Kamat et al., 2016 ▶). Several studies have shown promising neuroprotective properties as evidenced by its anticholinesterase activity, decrease of brain lipid peroxidation along with reducing thiobarbituric acid reactive substances (TBARS), and restoration of the intrinsic antioxidant protection system, such as glutathione (GSH) and superoxide dismutase (SOD) (Batiha et al., 2020 ▶). 

The present study set out to examine the effects of *A. absinthium *on scopolamine-induced memory and learning impairment and brain tissue oxidative damage in adult rats. 

## Materials and Methods

**Animals care and drugs treatment**

Fifty adults male Wistar rats, with the age of ten weeks old (230±20 g), were gotten from the animal care center of the School of Medicine, Mashhad University of Medical Sciences, Mashhad, Iran, and used in this research. They were kept in 4–5 per standard cages at room temperature (22±2°C), with a 12 hr light/dark cycle. The rats were provided with adequate food and water. The methods were approved by the Committee on Animal Research of Mashhad University of Medical Sciences (Ethical code: MUMS.MEDICAL.REC.1398.147). They were randomly divided into five groups (n=10 in each group) and treated for one week (Bora and Sharma, 2010 ▶; Marefati et al., 2019 ▶). Rats in group 1 (Control group) were administered with normal saline instead of the scopolamine and dimethyl sulfoxide (DMSO)/saline. Group 2 or Scopolamine group was administered with DMSO/saline only for one week and treated with scopolamine (2 mg/kg) 30 min before behavioral procedures assessments (Beheshti et al., 2021 ▶). Groups 3-5 were respectively treated daily with 50, 100 and 200 mg/kg of *A. absinthium *extract (Scopolamine-Ext 50, Scopolamine-Ext 100 and Scopolamine-Ext 200 groups) and injected with scopolamine (2 mg/kg, i.p.) before behavioral procedures assessments. On the final day, for investigation of antioxidant activity, the animals were anesthetized by urethane, and oxidant and antioxidant parameters in the cortex and hippocampus were measured. Scopolamine was bought from Sigma Aldrich Company, USA. Other materials needed for biochemical analysis were obtained from Merck Company**.**

**The plant extract**

The Herbarium of Khorasan Razavi Agricultural and Natural Resources Research Center provided* A. absinthium* with a voucher specimen (No. 11856). The method to prepare the hydroalcoholic extract of *A. absinthium* was described in former studies (Rakhshandeh et al., 2021 ▶; Rashidi et al., 2020 ▶)

**Morris water maze (MWM) test**

Spatial learning and memory were evaluated using the MWM. A circular black water pool (136 cm in diameter, 60 cm height, and 30 cm deep) containing water (24-26°C) was placed in a room with posters and computer hardware as visual cues. A translucent circular escape platform, 10 cm in diameter, was immersed 2 cm below the water surface in the center of the southwest quadrant and remained in that location throughout the experiment. In the beginning, the animals were exposed to the water maze for 60 sec without any chance to escape. The rats completed four trials for five days before the probe test. For each trial session, the animals were placed in the pool and permitted to swim till they reached the platform and rested on it for 15 sec. Each animal was released in four different positions towards the sidewall in a randomly chosen starting position [the boundaries of the four quadrants, labeled north (N), south (S), east (E), and west (W)]. After 60 sec, if an animal did not reach the hidden stage, it was directed for staying on it for 15 sec. After being taken out of the pool, the animals were dried and placed for five minutes in holding cages. An auto vision system was used to record the length of the swimming path and the time to reach the escape platform. Finally, the probe trial, consisting of a 60-sec free swimming test without an escape platform, was conducted on the sixth day. The distance traveled and the time elapsed in the target area were measured through the probe analysis (Karimi et al., 2015 ▶; Marefati et al., 2019 ▶).

**Passive avoidance (PA) test**

The PA learning test is based on negative strengthening and is used to evaluate non-spatial learning and memory. Along this test, rats learn to evade situations in which a harmful stimulus occurs, such as a gentle foot shock. The apparatus had two chambers, one dark and one light, with a grid ground connected by a small door (Akbari et al., 2019 ▶; Karimi et al., 2017 ▶). In the training session, the animals were positioned separately in the light side, facing away from the guillotine door, and were allowed to cross freely for 300 sec. On the second day, the rats were permitted to arrive into the dark chamber, and the gate was quietly closed. Then, they received 0.2 mA shock through the grid floor for 2 sec in the dark segment. During training sessions, the animals learned the associative rule that dark segment equals shock. In the test session, 3, 24, 48, and 72 hr after the shock, to evaluate their learning and memory, the animals were placed back into the apparatus without being shocked. After coming up the gate, they had access to the dark section for 300 sec. The rats with normal learning and memory avoid going into the section where they were once shocked. The time lag to cross the gate between the sections, the number of entries to the dark side, along with the time consumed in each section were measured and referred to as the retaining trial (Hakimi et al., 2019 ▶).

**Biochemical measurements**

 Afterward performing the behavioral studies, the animals were euthanized by i.p. administration of urethane (1.5 g/kg) (Hosseini et al., 2015 ▶). The cortical and hippocampal tissues were disconnected on an ice-cold superficial and kept at −80°C for subsequent proceeding. For biochemical measurements, the tissues were weighed and homogenized using phosphate buffered solution (PBS). Then, the homogenates were centrifuged at 1500 rpm for 10 min for scaling malondialdehyde (MDA) concentration, NO metabolites, total thiol content, SOD, and catalase (CAT) activities in both hippocampal and cortical tissues as described in prior studies (Akbari et al., 2019 ▶; Marefati et al., 2019 ▶). 

**Statistical analysis**

Data are presented as mean±standard error of the mean (SEM). One-way analysis of variance (ANOVA) was done for all parameters, followed by Tukey's *post hoc* comparisons test. A p<0.05 is described as statistical significance. Data management and analysis were performed using SPSS 16.0 (SPSS Inc., Chicago, IL, USA).

## Results

**Behavioral results**

***A. absinthium*** **improved scopolamine-induced memory impairment in the MWM test**

**Figure 1:**
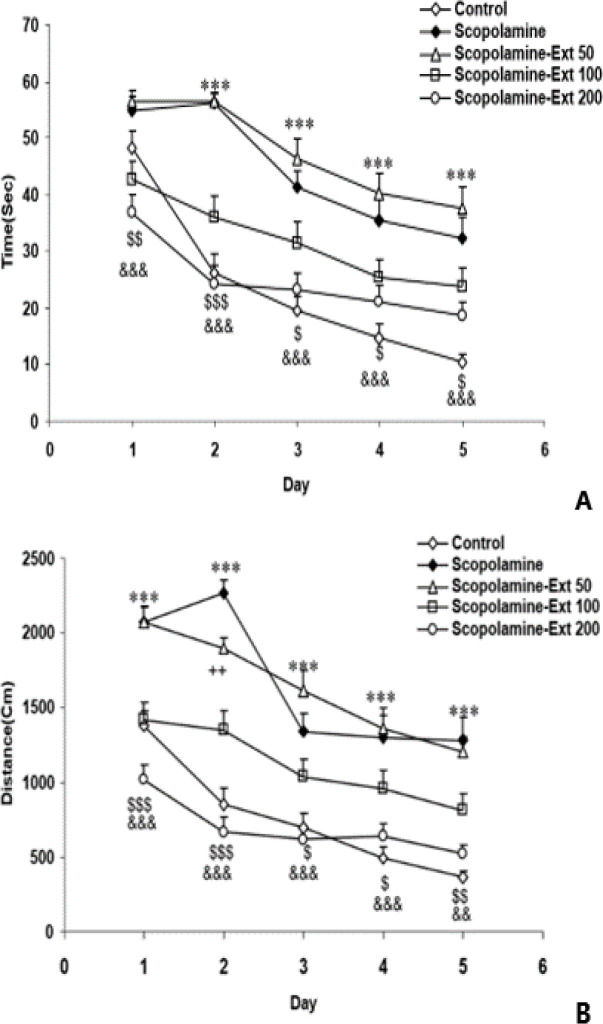
The delay time (A) and the distance traveled (B) to reach the hidden platform on five-day trials of the MWM task. The data are shown as mean±SEM, with n=10 in each group. ***p<0.001 depicts the difference between the Scopolamine and Control groups, ^++^p<0.01 depicts the difference between the Scopolamine-Ext 50 and Scopolamine animals,^ $^p<0.05,^ $$^p<0.01 and ^$$$^p<0.001 depict the difference between the Scopolamine-Ext 100 and Scopolamine groups, ^&&^p<0.01 and ^&&&^p<0.001 depict the difference between the Scopolamine-Ext 200 and Scopolamine groups. * depicts the difference between the Scopolamine and Control groups. (* depicts the difference between the Scopolamine and Control group, ^+^ depicts the difference between the Scopolamine-Ext 50 and Scopolamine animals, ^$^ depict the difference between the Scopolamine-Ext 100 and Scopolamine group, ^&^ depict the difference between the Scopolamine-Ext 200 and Scopolamine group) MWM: Morris water maze

The time latency and the distance traveled to reach the escape platform in the scopolamine group were significantly higher than those of the control animals (p<0.001 for both). Treatment with *A. absinthium* at all doses, 50, 100, and 200 mg/kg, decreased the latency time and the traveled distance in comparison with the scopolamine group (p<0.05- p<0.001). There were no significant differences among the three doses of *A. absinthium* (Figures 1A and 1B).

**Figure 2:**
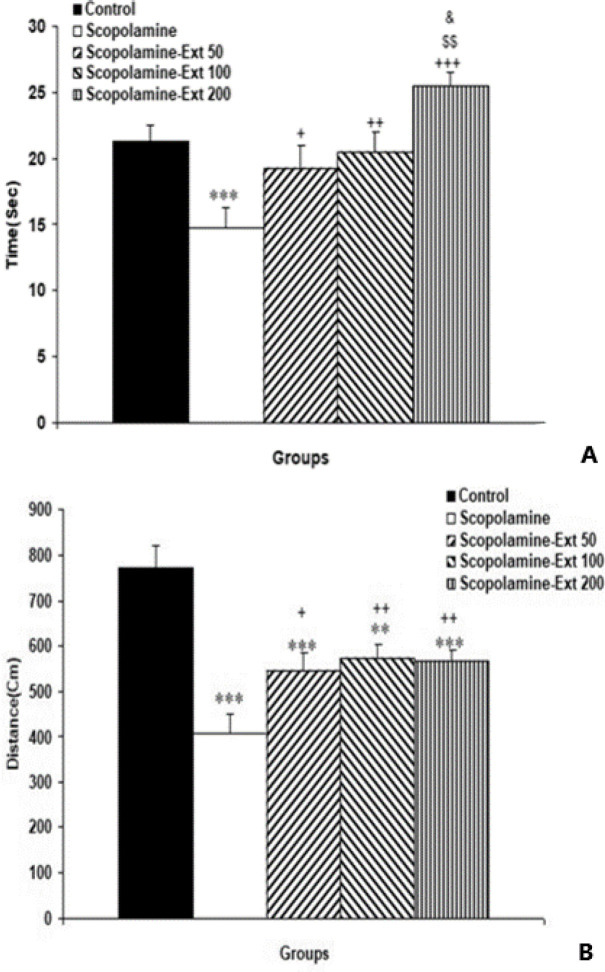
The time elapsed (A) and the distance traveled (B) in the target quadrant during the probe trial of the MWM task. The data are shown as mean±SEM, with n=10 in each group. **p<0.01 and ***p<0.001 compared with the Control animals, ^+^p<0.05,^ ++^p<0.01 and ^+++^p<0.001 compared with the Scopolamine animals,^ $$^p<0.01 compared with the Scopolamine-Ext 50 group^, &^p<0.05 compared with the Scopolamine-Ext 100 group. (* compared with the Control animals, ^+^ compared with the Scopolamine animals, ^$^ compared with the Scopolamine-Ext 50 group, ^&^ compared with the Scopolamine-Ext 100 group) MWM: Morris water maze

Probe trial data showed that the traveled distance and the elapsed time in the target quadrant in the Scopolamine group were lower compared to the control rats (p<0.001). It was found that all doses of *A. absinthium* improved the ability of animals to find the platform's location, as indicated by further traveled distance and longer time elapsed in the target area (p<0.05- p<0.01). However, the Scopolamine-Ext 200 group spent longer time in the target area than Scopolamine-Ext 50 and Scopolamine-Ext 100 animals (p<0.01 and p<0.05, respectively) (Figures 2A and 2B). 

***A. absinthium*** **improved scopolamine-induced memory impairment in the PA test**

The administration of scopolamine disrupted learning and memory as reflected by reduced latency time 3, 24, 48, and 72 hr following the shock compared with the Control animals (p<0.01- p<0.001). The results also showed that administration of *A. absinthium* at all doses prolonged the latency period at all post-shock times (p<0.05-p<0.001). 

**Figure 3:**
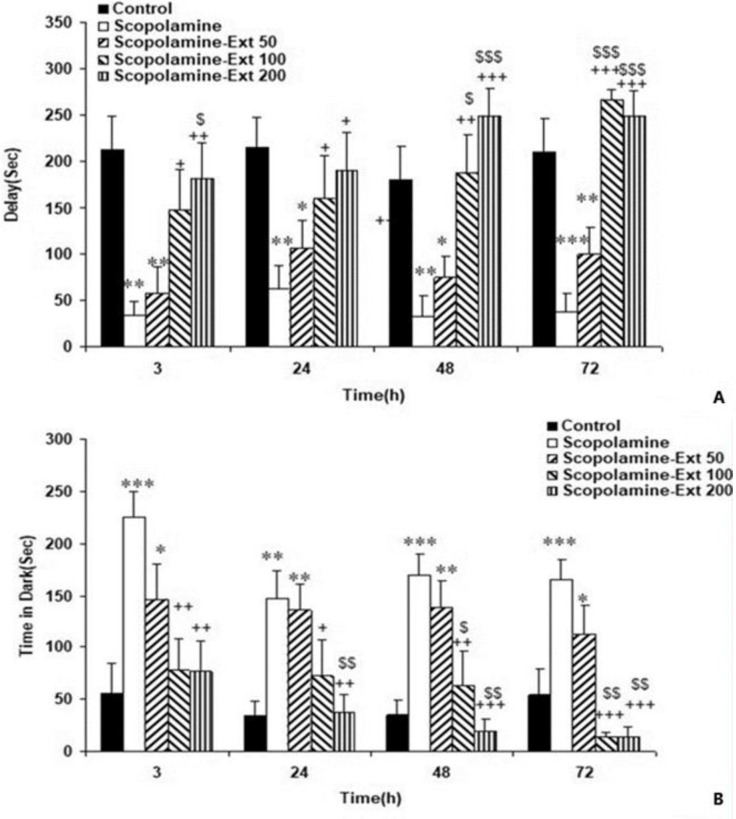
The delay time to pass in the dark component (A) and the dark time elapsed (B) 3, 24, 48 and 72 hr following the shock in the PA task. The data are shown as mean±SEM, with n=10 in each group. *p<0.05, **p<0.01 and ***p<0.001 compared to the Control animals, ^+^p<0.05,^ ++^p<0.01 and ^+++^p<0.001 compared to the Scopolamine animals, ^$^p<0.05,^ $$^p<0.01 and ^$$$^p<0.001 compared to the Scopolamine-Ext 50 group. (* compared with the Control animals, ^+^ compared with the Scopolamine animals, ^$^ compared with the Scopolamine-Ext 50 group, ^&^ compared with the Scopolamine-Ext 100 group) PA: Passive avoidance

Furthermore, the two higher doses of *A. absinthium* were more effective in reducing latency time than the 50 mg/kg dose (p<0.05-p<0.001). No significant difference was found between the two greater doses (Figure 3A).

**Figure 4:**
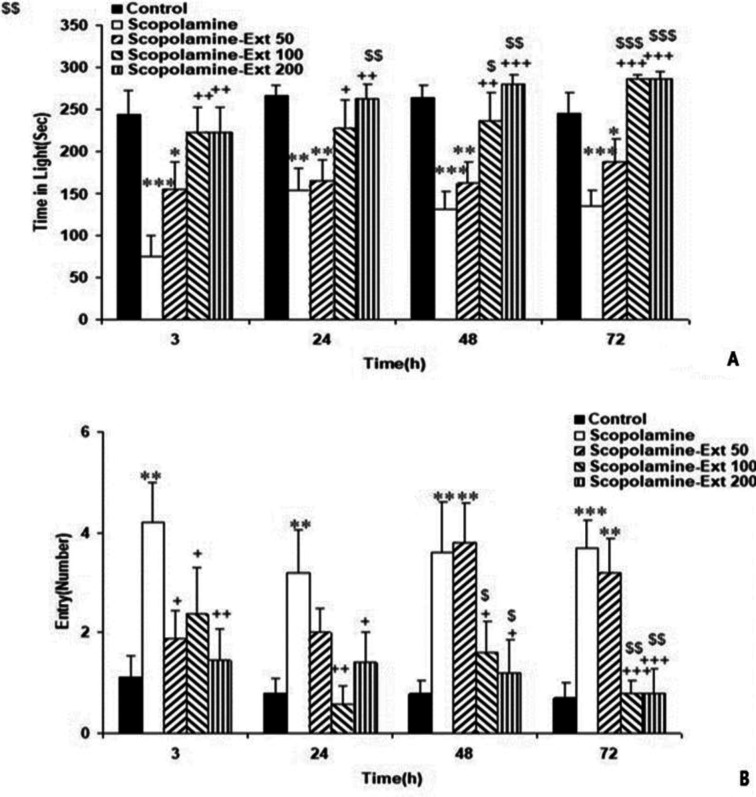
The light time elapsed (A) and the total number of entrances (B) in animal groups 3, 24, 48 and 72 hr following the shock in the PA task. The data are shown as mean±SEM, with n=10 in each group. *p<0.05, **p<0.01 and ***p<0.001 compared to the Control animals, +p<0.05, ++p<0.01 and +++p<0.001 compared to the Scopolamine animals, $p<0.05, $$p<0.01 and $$$p<0.001 compared to the Scopolamine-Ext 50 group. (* compared with the Control animals, ^+^ compared with the Scopolamine animals, ^$^ compared with the Scopolamine-Ext 50 group, ^&^ compared with the Scopolamine-Ext 100 group) PA: Passive avoidance

Scopolamine also prolonged dark time compared to the Control 3, 24, 48, and 72 hr following the shock (p<0.01-p<0.001). *A. absinthium* at all doses reversed the harmful effects of scopolamine, which reduced the dark time compared with the Control animals at post-shock delivery times (p<0.05-p<0.001). Additionally, 100 and 200 mg/kg of *A. absinthium* were both more effective than 50 mg/kg of the extract in reducing the time elapsed in the dark (p<0.05-p<0.01). But, the two greater doses were not significantly different (Figure 3B). Furthermore, scopolamine decreased the time passed in the light side compared to the Control group at all times following the shock (p<0.01-p<0.001). A. absinthium increased the time elapsed in the light section in all Scopolamine-Ext groups at all post-shock times (p<0.05-p<0.001). Furthermore, the two higher doses of the extract were more effective than the lowest dose (p<0.05- p<0.001). However, the light time in the Scopolamine-Ext 100 and 200 groups was not significantly different (Figure 4A).

Scopolamine also increased the total number of entrances 3, 24, 48, and 72 hr after the shock compared to the Control animals (p<0.01-p<0.001). The animals of the Scopolamine-Ext 50, 100, and 200 groups had lower number of entrances compared with the Scopolamine ones at all post-delivery shock times (p<0.05-p<0.001). Furthermore, the two greater doses of *A. absinthium* were more effective in decreasing the number of entrances compared with 50 mg/kg dose (p<0.05-p<0.01). However, the two higher doses were not significantly different (Figure 4B).

**Biochemical results**

***A. absinthium*** **decreased MDA and NO metabolites in the hippocampus**

**Figure 5:**
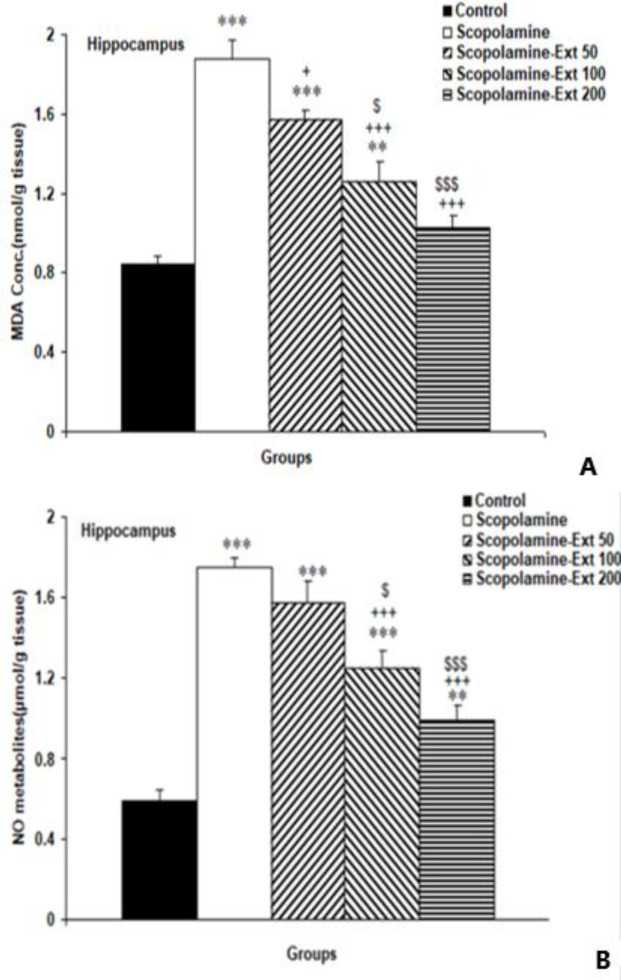
MDA concentration (A) and NO metabolites (B) in the hippocampal tissues. The data are shown as mean±SEM, with n=10 in each group. **p<0.01 and ***p<0.001 compared with the Control animals, ^+^p<0.05 and ^+++^p<0.001 compared with the Scopolamine animals, ^$^p<0.05 and ^$$$^p<0.001 compared with the Scopolamine-Ext 50 group. (* compared with the Control animals, ^+^ compared with the Scopolamine animals, ^$^ compared with the Scopolamine-Ext 50 group, ^&^ compared with the Scopolamine-Ext 100 group) A=MDA: malondialdehyde, B=NO metabolites: nitric oxide metabolites

The biochemical data indicated that scopolamine-induced neuroinflammation was presented by increased hippocampal MDA (p<0.001). *A. absinthium* at all doses decreased MDA concentrations compared to the Scopolamine animals (p<0.05- p<0.001). Furthermore, both higher doses, 100 and 200 mg/kg, of the extract had a higher efficacy in decreasing the level of MDA compared with the lowest dose (p<0.05 and p<0.001, respectively). But the two greater doses were not significantly different (Figure 5A).

The findings also showed that injection of scopolamine increased NO metabolites in the hippocampal tissue compared to the Control animals (p<0.001).

*A. absinthium* decreased NO metabolites in Scopolamine-Ext 100 and 200 groups (both p<0.001). But its lowest dose (50 mg/kg) was not significantly more effective than the Scopolamine group because has no significant difference compare to scopolamine. Moreover, 100 and 200 mg/kg of *A. absinthium* were more effective in decreasing NO metabolites than its lowest dose (p<0.05 and p<0.001, respectively). No significant difference was noted between the medium and highest doses (Figure 5B).

***A. absinthium*** **improved total thiol content and CAT and SOD activities in the hippocampus**

**Figure 6:**
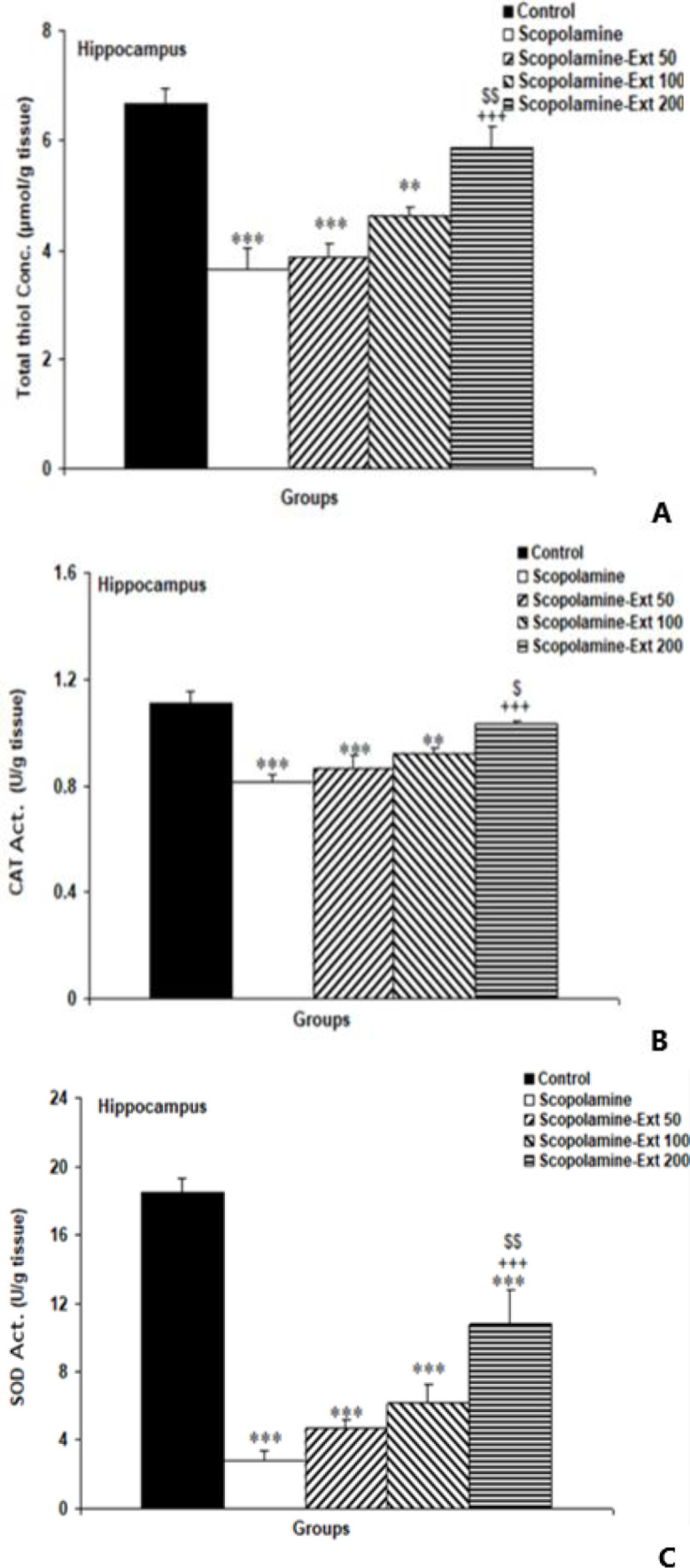
Total thiol content (A) and CAT (B) and SOD (C) activities in the hippocampal tissues. The data are shown as mean±SEM, with n=10 in each group. **p<0.01 and ***p<0.001 compared with the Control animals, ^+++^p<0.001 compared with the Scopolamine animals, ^$^p<0.05 and^ $$^p<0.01 compared with the Scopolamine-Ext 50 group. (* compared with the Control animals, ^+^ compared with the Scopolamine animals, ^$^ compared with the Scopolamine-Ext 50 group, ^&^ compared with the Scopolamine-Ext 100 group) B=CAT: catalase, C=SOD: superoxide dismutase

The biochemical assessments revealed that scopolamine decreased anti-oxidant biomarkers, including total thiol content, CAT, and SOD, compared to the Control animals (p<0.001 for all). The highest dose of the extract (200 mg/kg) increased total thiol, SOD, and CAT in comparison to the scopolamine group (p<0.001 for all). Interestingly, it was more effective than the 50 mg/kg dose in decreasing total thiol content, SOD (p<0.01; Figures 6A, C), and CAT (p<0.05; Figure 6B). But the lowest and medium doses could not significantly improve the level of anti-oxidant biomarkers (Figure 6). 

***A. absinthium*** **decreased MDA and NO metabolites in the cortex**

**Figure 7:**
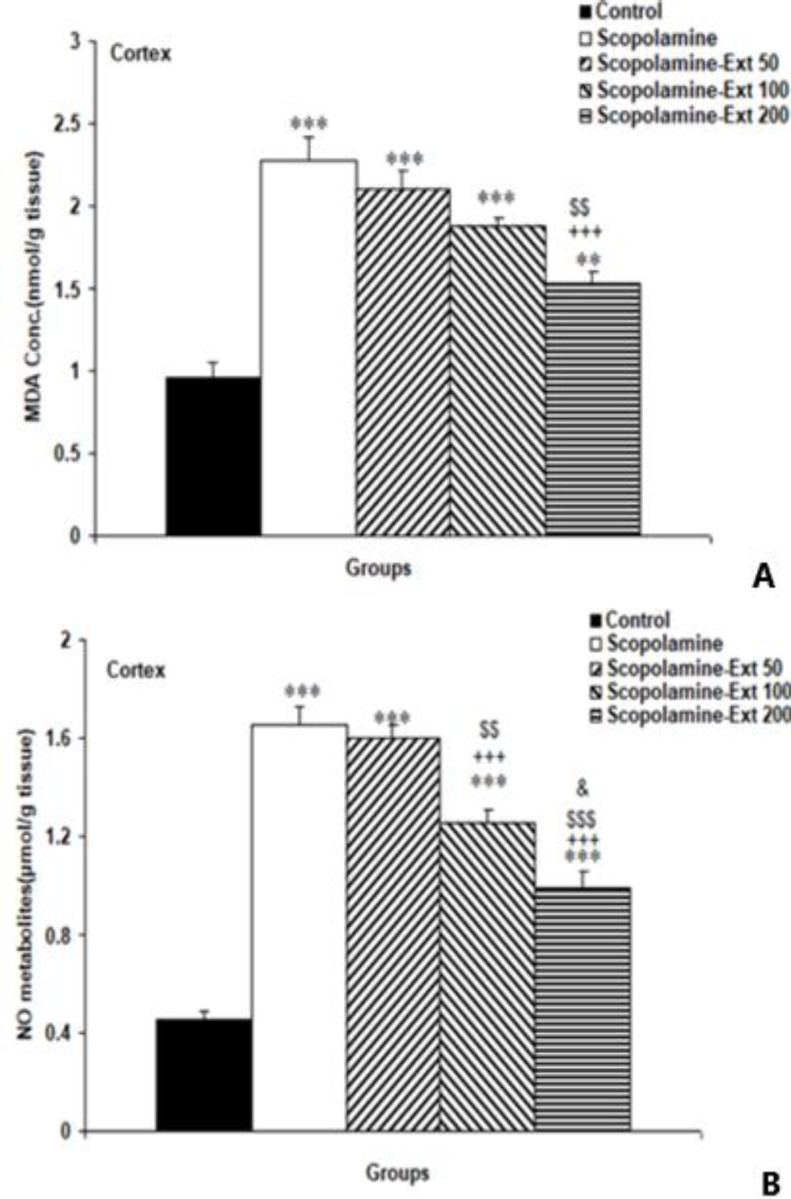
MDA concentration (A) and NO metabolites (B) in the cortex. The data are shown as mean±SEM, with n=10 in each group. **p<0.01 and ***p<0.001 compared with the Control animals, ^+++^p<0.001 compared with the Scopolamine animals, ^$$^p<0.01 and ^$$$^p<0.001 compared with the Scopolamine-Ext 50 group, ^&^p<0.05 compared with the Scopolamine-Ext 100 group. (* compared with the Control animals, ^+^ compared with the Scopolamine animals, ^$^ compared with the Scopolamine-Ext 50 group, ^&^ compared with the Scopolamine-Ext 100 group) A=MDA: malondialdehyde, B=NO metabolites: nitric oxide metabolites

The biochemical data also indicated that scopolamine-induced neuroinflammation led to a higher cortical MDA compared to the Control group (p<0.001). As compared to the Scopolamine group and the Scopolamine-Ext 50 group, pretreatment with 200 mg/kg of *A. absinthium* decreased MDA levels in the cortex (p<0.001 and p<0.01, respectively). But *A. absinthium* at 50 and 100 mg/kg could not attenuate cortical MDA significantly (Figure 7A). Scopolamine increased cortical NO metabolites compared to the Control group (p<0.001). NO metabolites of the Scopolamine-Ext 100 and Scopolamine-Ext 200 groups were significantly less than those of the Scopolamine group (both p<0.001). However, *A. absinthium* 50 mg/kg was not able to reduce cortical NO metabolites. Additionally, cortical NO metabolites in the animals treated with *A. absinthium* 100 mg/kg and 200 mg/kg were lower than the Scopolamine-Ext 50 group (p<0.01 and p<0.001, respectively). Treating with 200 mg/kg of *A. absinthium* was more efficient than 100 mg/kg in attenuating cortical NO metabolites (p<0.05) (Figure 7B).

***A. absinthium*** **improved total thiol content and CAT and SOD activities in the cortex**

**Figure 8:**
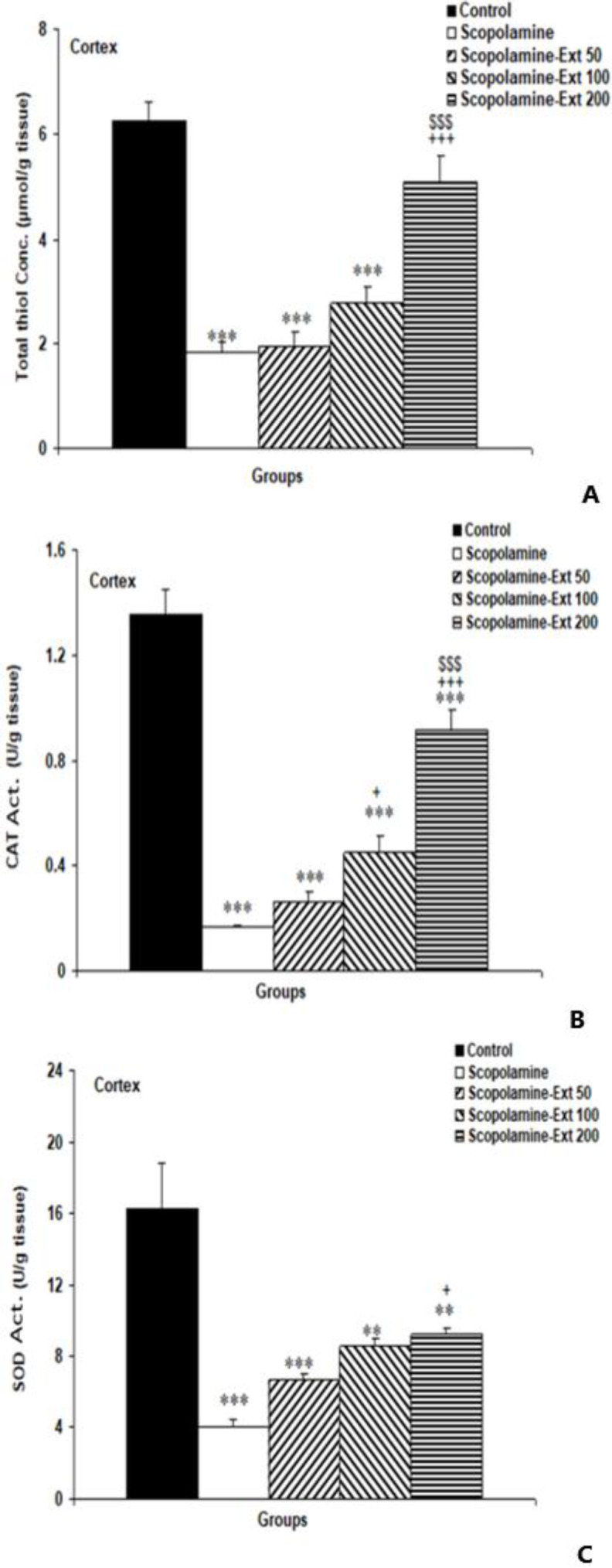
Total thiol content (A) and CAT (B) and SOD (C) activities in the cortex. The data are shown as mean±SEM, with n=10 in each group. **p<0.01 and ***P<0.001 compared with the Control animals, +p<0.05 and +++p<0.001 compared with the Scopolamine animals, $$$p<0.001 compared with the Scopolamine-Ext 50 group. (* compared with the Control animals, ^+^ compared with the Scopolamine animals, ^$^ compared with the Scopolamine-Ext 50 group, ^&^ compared with the Scopolamine-Ext 100 group) B=CAT: catalase, C=SOD: superoxide dismutase

In the cortical tissue, treatment with scopolamine decreased total thiol content (p<0.001). The Scopolamine-Ext 200 animals had significantly greater total thiol content than the Scopolamine-Ext 50 and Scopolamine groups (both p<0.001). However, no significant improvement in total thiol was observed between animals treated with 50 and 100 mg/kg of the extract (Figure 8A).

Based on our results, cortical CAT was decreased due to scopolamine administration (p<0.001). In comparison to the Scopolamine animals, *A. absinthium* at the two higher doses, 100 and 200 mg/kg, enhanced CAT activity in cortical tissues (p<0.05 and p<0.001, respectively). However, the extract was not effective at 50 mg/kg. It was found that rats treated with 200 mg/kg of *A. absinthium* had higher CAT activities than those treated with 50 mg/kg (p<0.001). But the differences between the Scopolamine-Ext 50 and 100 were not statistically significant (Figure 8B). 

Scopolamine also inhibited SOD activity (p<0.001), and this effect was restored by 200 mg/kg of *A. absinthium* (p<0.05). Compared with the Scopolamine rats, there was no improvement in cortical SOD with the lowest and medium doses of the extract. Moreover, no significant difference in SOD activities was observed among the three doses of *A. absinthium *extract (Figure 8C).

## Discussion

According to the results of this study, *A. absinthium* improved spatial and passive avoidance memory impairment induced by scopolamine by ameliorating the brain tissue oxidative stress criteria.

Scopolamine is a non-selective anti-muscarinic agent which can cross the blood-brain barrier, causing a dementia model (Hafez et al., 2017 ▶). The current study, like previous studies, confirmed that scopolamine has detrimental effects on spatial learning and memory in the MWM test (Deepa et al., 2020 ▶; Mostafa, 2018 ▶; Ngoupaye et al., 2017 ▶). It also disrupts the cognitive performances of the animals in the PA test (Rabiei and Setorki, 2018 ▶; Rahnama et al., 2015 ▶; Upadhyay et al., 2018 ▶). Scopolamine through blocking acetylcholine (ACh) muscarinic receptors, affects ACh release, resulting in neuronal apoptosis, hippocampus damage and impaired learning and memory (Jafarian et al., 2019 ▶; Sodhi et al., 2014 ▶). It was also demonstrated that memory impairment induced by scopolamine is linked to increased brain lipid peroxidation and decreased antioxidant potential within the brain (Zaki et al., 2014 ▶). The present study also showed that scopolamine increased MDA and NO metabolites as oxidative stress factors and decreased antioxidant defense system factors like SOD, CAT and total thiol content. 

Based on our results, *A. absinthium* improved scopolamine induced-memory and learning impairment, as evidenced by reduced traveled distance and latency time to find the hidden platform in the MWM task. All three treatment groups, especially the highest dose, spent more time and traveled a greater distance in the target region than the Scopolamine group (indicating dose-dependency), which suggested that these rats had a greater ability to recall the platform's location. Additionally, administration of *A. absinthium* at all doses resulted in a significant dose-dependent improvement in PA tasks. 

There are no documented effects of *A. absinthium* on scopolamine-induced memory impairment to compare with the findings of our study; nevertheless, using other behavioral tests, the potential benefits of *A. absinthium* have been reported. Bora et al. (2010) ▶ revealed that oral administration of *A. absinthium* (100 mg/kg and 200 mg/kg) improved the elevated plus maze (EPM) task which was used for the assessment of short-term memory impairment following cerebral ischemia and reperfusion injury in rats. Additionally, aqueous extract of *A. absinthium* (100 and 200 mg/kg, p.o.) has been shown to improve the parameters of open field test (OFT) and reduce neurotoxicological damage induced by Aluminum (Kharoubi et al., 2016 ▶).

Using EPM and OFT paradigms, it was also shown that* A. absinthium* (300 mg/kg, p.o.) modified cognitive impairments induced by prolonged exposure to lead (Amel et al., 2016 ▶). Also, other Asteraceae species such as *A. persica *and *A. princeps *var.* orientalis *reduced the detrimental effect of scopolamine on learning and memory dysfunction in the MWM and PA tasks (Liu et al., 2012 ▶; Rabiei and Setorki, 2020 ▶).

There is a close connection between learning and memory dysfunction, oxidative stress, and neuronal damage (Ghasemi et al., 2019 ▶). As a result of high oxygen consumption and poor antioxidant capacity, brain tissue is more likely to be affected by oxidative damage (Budzynska et al., 2015 ▶). Reactive oxygen species (ROS) promote lipid peroxidation, leading to neuronal degeneration, especially in central cholinergic pathways (Ishola et al., 2017 ▶). Considering this, antioxidants may be useful for the treatment of memory dysfunction by reducing or preventing oxidative damage (Kim et al., 2018 ▶). *A. absinthium* has previously been claimed to have protective effects against oxidative stress in the brain. It has been demonstrated that methanolic extract of *A. absinthium* possesses neuroprotective properties, as evidenced by a decline in lipid peroxidation associated with the reduction of TBARS levels and restoration of endogenous antioxidant enzymes such as GSH and SOD (Bora and Sharma, 2011 ▶). Another study showed that oral administration of *A. absinthium* reduced oxidative stress caused by mercury (II) chloride (HgCl_2_), which was associated with a decrease in MDA levels in the brain and recovery of antioxidant enzyme activities including CAT, SOD, glutathione reductase, and glutathione peroxidase (GPx) (Hallal et al., 2016 ▶). Besides, treatment with *A. absinthium* (50 mg/kg) could reduce oxidative damage by increasing serum levels of total thiol (Mohammadian et al., 2016 ▶).

It has also been documented that NO affects memory and learning processes. NO is an important neuromodulator in neuronal signaling, promoting synaptic plasticity in various brain regions, including the cerebrum and hippocampus. NO enhances synaptic plasticity at physiological concentrations, but when its overproduction, it becomes toxic and impairs learning and memory (Babaei et al., 2012 ▶; Hosseini et al., 2018 ▶). In neurodegenerative disorders, the reaction between NO and superoxide in the formation of peroxynitrite is considered one of the most critical pathways leading to DNA damage and neuronal death. NO overproduction is also responsible for disrupting a number of biochemical processes including those involving lipid peroxidation, oxidation of protein, and thiol oxidation (Anaeigoudari et al., 2016 ▶). Bora et al. (2011) ▶ documented the *in vitro* antioxidant effect of *A. absinthium.* The methanolic extract of *A. absinthium* exhibited nitric oxide scavenging activity in a concentration-dependent manner between 25 and 200 g/ml (EC_50_ = 180.71 μg/ml). Lee et al. (2004) revealed that 5, 6, 30, 50-tetra methoxy 7, 40-hydroxy flavone isolated from *A. absinthium *inhibited both inducible NO synthase (iNOS) expression and NO release in lipopolysaccharide-stimulated RAW 264.7 cells. As well, cardomonin obtained from *A. absinthium* suppressed the expression of iNOS and NO production through its direct effect upon transcription factors binding to DNA (Hatziieremia et al., 2006 ▶). 

These findings are in accord with our results indicating that *A. absinthium* administration decreased MDA and NO metabolites while augmented CAT and SOD activities, and total thiol content in the cortex and hippocampus. Notably, the effect of the extract on the cortical SOD was not dose-dependent, whereas its effect on other oxidant and antioxidant biomarkers in both hippocampal and cortical tissues was dose-dependent. These effects may provide new therapeutic opportunities for the control of neuroinflammatory disorders with excessive oxidative stress.

This study showed that administration of *A. absinthium* improved scopolamine-induced learning and memory impairments in both MWM and PA tasks. The effects were potentially mediated by ameliorating the oxidative damage in the brain tissues. Future studies regarding the effects of* A. absinthium* on the nervous system would be worthwhile

## Conflicts of interest

The authors have declared that there is no conflict of interest.
